# Changes in sedentary behavior patterns during the transition from childhood to adolescence and their association with adiposity: a prospective study based on compositional data analysis

**DOI:** 10.1186/s13690-021-00755-5

**Published:** 2022-01-04

**Authors:** Lukáš Rubín, Aleš Gába, Jana Pelclová, Nikola Štefelová, Lukáš Jakubec, Jan Dygrýn, Karel Hron

**Affiliations:** 1 Faculty of Physical Culture, Palacký University Olomouc, Olomouc, Czech Republic; 2 Faculty of Science, Humanities and Education, Technical University of Liberec, Liberec, Czech Republic; 3 Faculty of Science, Palacký University Olomouc, Olomouc, Czech Republic

**Keywords:** Movement behaviors, Physical activity, Sedentary lifestyle, Prolonged sitting, Fat mass, Fat mass index, Visceral adipose tissue, Child, Adolescent

## Abstract

**Background:**

To date, no longitudinal study using a compositional approach has examined sedentary behavior (SB) patterns in relation to adiposity in the pediatric population. Therefore, our aims were to (1) investigate the changes in SB patterns and adiposity from childhood to adolescence, (2) analyze the prospective compositional associations between changes in SB patterns and adiposity, and (3) estimate the changes in adiposity associated with substituting SB with physical activity (PA) of different intensities.

**Methods:**

The study presents a longitudinal design with a 5-year follow-up. A total of 88 participants (61% girls) were included in the analysis. PA and SB were monitored for seven consecutive days using a hip-worn accelerometer. Adiposity markers (fat mass percentage [FM%], fat mass index [FMI], and visceral adiposity tissue [VAT]) were assessed using the multi-frequency bioimpedance analysis. The prospective associations were examined using compositional data analysis.

**Results:**

Over the follow-up period, the proportion of time spent in total SB increased by 154.8 min/day (*p* < 0.001). The increase in total SB was caused mainly by an increase in middle and long sedentary bouts, as these SB periods increased by 79.8 min/day and 62.0 min/day (*p* < 0.001 for both), respectively. FM%, FMI, and VAT increased by 2.4% points, 1.0 kg/m^2^, and 31.5 cm^2^ (*p* < 0.001 for all), respectively. Relative to the remaining movement behaviors, the increase in time spent in middle sedentary bouts was significantly associated with higher FM% (*β*_ilr1_ = 0.27, 95% confidence interval [CI]: 0.02 to 0.53) at follow-up. Lower VAT by 3.3% (95% CI: 0.8 to 5.7), 3.8% (95% CI: 0.03 to 7.4), 3.9% (95% CI: 0.8 to 6.9), and 3.8% (95% CI: 0.7 to 6.9) was associated with substituting 15 min/week spent in total SB and in short, middle, and long sedentary bouts, respectively, with an equivalent amount of time spent in vigorous PA.

**Conclusions:**

This study showed unfavorable changes in SB patterns and adiposity status in the transition from childhood to adolescence. Incorporating high-intensity PA at the expense of SB appears to be an appropriate approach to reduce the risk of excess adiposity in the pediatric population.

**Supplementary Information:**

The online version contains supplementary material available at 10.1186/s13690-021-00755-5.

## Background

The growing percentage of children who are classified as overweight or obese has reached an epidemic level worldwide and is considered one of the most serious public health challenges [1]. The number of children with an unhealthy weight has more than quadrupled in the past four decades [2], which has resulted in the current estimate of approximately one-fifth of youth being overweight or obese [3]. Scientific evidence has shown that overweight and obesity tend to trend upward from childhood to adolescence [4, 5] and, by extension, until adulthood [5, 6]. Excess adiposity leads to the development of several non-communicable diseases across the lifespan [7, 8] and has serious consequences such as premature mortality and physical morbidity in adulthood [9].

An increased accumulation of body fat is closely related to the way children spend their daily time. Previous studies have shown that unhealthy time-use characterized by an excessive amount of time spent in sedentary behavior (SB) at the expense of physical activity (PA) is associated with excess adiposity [10–12]. SB is a complex movement behavior of low energy expenditure (≤1.5 metabolic equivalents) and includes various behaviors occurring in different body postures (i.e., sitting, reclining, and lying down) [13]. In addition to total sedentary time, adiposity status is also associated with patterns of how SB accumulates. Since unfavorable adiposity status is associated with prolonged uninterrupted SB rather than with total SB [14], patterns of accumulation should be considered when investigating the role of SB in obesity prevention.

SB is considered a predominant component of a 24-h daily cycle. Therefore, compositional data analysis (CoDA) is recommended to find the optimal time-use for obesity prevention [15–17]. This analytical approach also allows the estimation of a theoretical change in adiposity resulting from substituting SB with other daily movement behaviors. Previous CoDA-based studies suggested that an effective strategy to control and prevent childhood obesity is to increase moderate-to-vigorous physical activity (MVPA) at the expense of total and prolonged SB [10, 14, 18] and that adiposity status could also be improved by substituting longer sedentary bouts with shorter sedentary bouts [14]. Such estimates provide a guide for developing intervention strategies for obesity prevention and help existing interventions scale up their effectiveness.

To deepen the understanding of the role of SB in the prevention of excess adiposity, it appears important to describe changes in SB patterns during the transition from childhood to adolescence. It has been documented that an increase in total SB in this life period is caused primarily by prolonged uninterrupted SB [19, 20]. Such age-related changes in daily time use may detrimentally influence adiposity when children grow older. However, evidence suggesting an optimal change in SB patterns for healthy adiposity during the transition from childhood to adolescence is lacking. To our knowledge, no CoDA-based study has estimated the effect of longitudinal changes in SB patterns in the pediatric population on adiposity status. Therefore, the main objectives of this study were to (1) investigate the longitudinal changes in SB patterns and adiposity markers from childhood to adolescence, (2) analyze the prospective compositional associations between changes in SB patterns from childhood to adolescence and adiposity, and (3) estimate the changes in adiposity associated with substituting SB with PA of different intensities.

## Methods

### Participants

Study participants were pupils from public elementary schools located in the eastern part of the Czech Republic (Moravia region). A total of 24 elementary schools were asked to participate in the study, 8 of which agreed. Sport schools (academies) or schools with special educational needs were not included. Data were intentionally collected in the similar spring and fall seasons from April 2013 to May 2014 for the baseline period and from April 2018 to May 2019 for the follow-up period to avoid any inconsistencies in weather conditions that could affect the movement behaviors of the participants.

The main inclusion criterion for study participants was good health as reported by parents or guardians. Participants with medical conditions that could affect engagement in habitual PA and/or adiposity were not included in the study. The study objectives and content of the research study were presented to the parents or guardians using an information booklet. The parents or guardians were also given a telephone number to inquire about any additional information or to clarify the objective and extent of the study. A total of 311 children participated in the study at baseline. Out of these, 88 participants (61% girls) provided complete data for all variables of interest and were included in the final sample. A total of 223 participants were excluded from the study for various reasons (e.g., provided incomplete data, withdrawal). The final sample size was sufficient to ensure statistical power of > 80% in the regression model with 9 explanatory variables for alpha error of 0.05 and assuming at least medium effect size (f^2^ ≥ 0.2) in the population, according to Cohen [21]. The characteristics of the final sample are presented in Table 1.

**Table 1:** Characteristics of participants at baseline and follow-up (*n* = 88)

	Baseline		Follow-up		Difference		
	Mean^a^	SD^b^	Mean^a^	SD^b^	Mean^a^	SD^b^	*p*-value^c^
Age (years)	9.2	0.9	14.6	0.8	5.4	0.3	**< 0.001**
Height (cm)	137.4	7.3	166.4	8.4	29.0	5.1	**< 0.001**
Weight (kg)	32.3	7.4	56.6	11.5	24.3	6.6	**< 0.001**
BMI *z*-score	0.18	1.19	0.04	1.07	−0.14	0.69	0.074
Fat mass (%)	16.2	8.3	18.6	8.6	2.4	6.1	**< 0.001**
Fat mass index (kg/m^2^)	2.9	2.1	4.0	2.4	1.1	1.6	**< 0.001**
Visceral adipose tissue (cm^2^)	27.4	23.4	45.3	31.5	17.9	21.8	**< 0.001**
**Weight status**^d^							
Underweight (%)	2.3		1.1		−1.2		1.000
Normal weight (%)	73.8		76.1		2.3		0.803
Overweight (%)	14.8		20.5		5.7		0.359
Obesity (%)	9.1		2.3		−6.8		**0.041**
**Movement behaviors (the 4-part composition)**							
Total SB (min/day)	328.2	49.8	483.0	44.6	154.8	41.5	**< 0.001**
LPA (min/day)	354.3	42.3	260.6	42.0	−93.7	39.4	**< 0.001**
MPA (min/day)	41.1	42.4	28.6	38.0	−12.5	38.1	**< 0.001**
VPA (min/day)	12.8	65.5	16.2	75.5	3.4	81.0	**0.005**
**Movement behaviors (the 6-part composition)**							
SB short bouts (min/day)	195.4	22.9	209.7	22.7	14.3	22.8	**< 0.001**
SB middle bouts (min/day)	101.5	28.6	181.3	24.4	79.8	27.7	**< 0.001**
SB long bouts (min/day)	36.5	47.9	98.5	42.8	62.0	45.4	**< 0.001**
LPA (min/day)	352.1	23.1	255.9	24.2	−96.2	24.7	**< 0.001**
MPA (min/day)	39.6	27.6	28.2	26.7	−11.4	27.1	**< 0.001**
VPA (min/day)	11.3	49.9	14.8	59.2	3.5	52.3	0.868

Bold values denote significant results

*LPA* light physical activity, *MPA* moderate physical activity, *SB* sedentary behavior, *SD* standard deviation, *VPA* vigorous physical activity

^a^The robust mean for compositional data; arithmetic mean for non-compositional continuous variables; percentages for categorical variables

^b^Part of the total variance related to a given time-use component for compositional data; standard deviation for other variables

^c^Analyzed using the paired sample t-test for continuous variables (the first pivot coordinate was used to represent each time-use variable for compositional data) and the chi-squared test for categorical variables

^d^Based on BMI *z*-score categories

### Anthropometric measurements and adiposity assessment

Each participant’s standing height was measured before the adiposity assessment using a standardized procedure via an anthropometer P-375 (Trystom, Olomouc, Czech Republic) with an accuracy of 0.1 cm. The body weight was measured to the nearest 0.1 kg via the device InBody 720 (InBody, Seoul, Korea). Sex- and age-standardized World Health Organization (WHO) body nass index (BMI) *z*-scores were calculated to categorize participants according to weight status [22].

The assessment of adiposity was performed by multi-frequency (1–1000 kHz) bioimpedance analysis using an InBody 720 body composition analyzer, which is considered highly precise for measuring body composition in the pediatric population [23]. Following the manufacturer’s guidelines, the measurement was performed in a standing position, barefoot, and in light indoor clothing. The participants were instructed to fast for at least 4 h and maintain proper hydration for at least 24 h prior to the examination to maintain the validity of the measurement. One field worker performed the measurement on the school premises. Adiposity was expressed as the fat mass percentage (FM%, %), fat mass index (FMI, kg/m^2^), and visceral adiposity tissue (VAT, cm^2^).

### Physical activity and sedentary behavior assessment

A hip-mounted ActiGraph GT3X accelerometer (ActiGraph, Pensacola, FL, USA) was used to assess PA and SB for seven consecutive days. A full description of the measurement protocol is provided elsewhere [24]. Briefly, participants were instructed to wear the device during the waking time period each day excluding time when they were in contact with water (e.g., bathing or swimming). Before monitoring, the accelerometers were initialized to collect data in 1 min intervals. Non-wear time was defined by an interval of at least 60 consecutive minutes of zero activity intensity counts [25], not allowing any interruptions [26]. For wear time data, SB was defined as 0–100 counts per minute (cpm), light PA (LPA) as 101–2295 cpm, moderate PA (MPA) as 2296–4011 cpm, and vigorous PA (VPA) as ≥ 4012 cpm [27]. The Evenson cut-off points provided an acceptable classification accuracy for all levels of PA intensity among children and adolescents [28]. In addition, these cut-off points are the most common cut-off points used in studies evaluating longitudinal changes in PA in children and adolescents [29]. A sedentary bout was defined as 1 or more consecutive minutes with fewer than 100 cpm. Sedentary bouts with durations of 1–9 min, 10–29 min, and ≥ 30 min, that is, short, middle, and long sedentary bouts, respectively, were analyzed. The thresholds for sedentary bouts were consulted based on the previous literature [30] and in order to minimize the number of zero values. Accelerometer data were considered valid if the participant wore the device for at least 4 days, including 1 weekend day, with ≥ 10 h of wear time per school day and ≥ 8 h of wear time per weekend day [31]. Only those participants who had valid accelerometer data for both baseline and follow-up were acceptable for analysis.

### Statistical analyses

Statistical analyses were conducted using IBM SPSS Statistics version 23 (IBM, Armonk, NY, USA) and R version 3.4.2 (R Foundation for Statistical Computing, Vienna, Austria). The differences between baseline and follow-up were analyzed using the paired sample *t*-test. The chi-squared test was used to compare proportions. One-way analysis of covariance adjusted for age and sex was used to perform a sensitivity analysis to assess differences between the participants included and excluded from the study.

CoDA was carried out to analyze differences in the time-use patterns between the baseline and follow-up movement behavioral data. The robust compositional mean and the variation matrix were calculated for the purpose of descriptive statistics [32] to reduce the influence of possible aberrant observations. To analyze the data, two wake-time compositions were created. The 4-part composition consisted of total SB, LPA, MPA, and VPA, and the 6-part composition consisted of total SB decomposed into bouts of different durations (i.e., short, middle, and long sedentary bouts), LPA, MPA, and VPA. Accordingly, the relative information about the other components (LPA, MPA and VPA) also necessarily changed between the compositions, as each of these components was determined by five log-ratios in the latter compostion instead of three in the former composition. A Bayesian-multiplicative approach [33] was used to replace zero values that occurred in long sedentary bouts in 3% of participants. The compositions were expressed in the pivot coordinates system, being a special case of isometric log-ratios (*ilr*s). From each of the coordinate systems assessed, only the first pivot coordinate (i.e., *ilr*_1_) was interpreted in all analyses.

A robust compositional regression analysis [32] was performed to analyze the prospective associations between the changes in SB patterns and adiposity. Based on our previous experience [34], we used regression models in which the differences between follow-up and baseline SB patterns (in terms of pivot coordinates) were set as the independent variables, and adiposity markers at follow-up were set as the dependent variables. The regression models were further adjusted for the corresponding dependent variables at baseline and the sex and age of the participants. Isotemporal substitution modeling was used to estimate the changes in different adiposity markers associated with one-to-one reallocation between SB and PA. For this purpose, wake-time compositions were closed to 16 h because of the assumption of an average of 8 h of sleep per day based on previous reports of sleep duration in the target population [35]. Estimated changes in adiposity markers were considered significant when 95% confidence intervals (CIs) did not cover zero. All statistical analyses were conducted at a significance level of *p* < 0.05.

## Results

A total of 311 participants with a mean age of 9.2 ± 1.0 years participated in the study at baseline. Of these, 135 participants attended the follow-up investigation. Of all participants who attended the follow-up investigation, 88 provided complete data for all variables of interest and were included in the final analysis. No significant differences in adiposity indicators were observed between participants who were included and excluded (Table S1). Participants who were included only had lower body height (*p* < 0.001) and weight (*p* = 0.006) than those who were excluded.

The characteristics of the study participants included in the final analysis are presented in Table 1. For the final sample, the mean follow-up duration was 5.0 ± 0.1 years. The median number of valid days was 7 days for both time points. Nearly 24% of participants were overweight and obese at baseline, and FM%, FMI, and VAT increased by 2.4% points, 1.1 kg/m^2^, and 17.9 cm^2^ (*p* < 0.001 for all), respectively, over the 5-year period. The proportion of time spent in all movement behaviors within the 4-part waking-time composition changed significantly over the follow-up period. Specifically, the proportion of time spent in total SB and VPA increased by 47% (difference: 154.8 min/day; *p* < 0.001) and 27% (difference: 3.4 min/day; *p* = 0.005), respectively, while the proportions of time spent in LPA and MPA decreased by 26% (difference: 93.7 min/day; *p* < 0.001) and 30% (difference: 12.5 min/day; *p* < 0.001), respectively. The increase in total SB was caused mainly by an increase in the middle and long sedentary bouts, as they increased by 79% (difference: 79.8 min/day; *p* < 0.001) and 170% (difference: 62.0 min/day; *p* < 0.001), respectively.

**Fig. 1:**
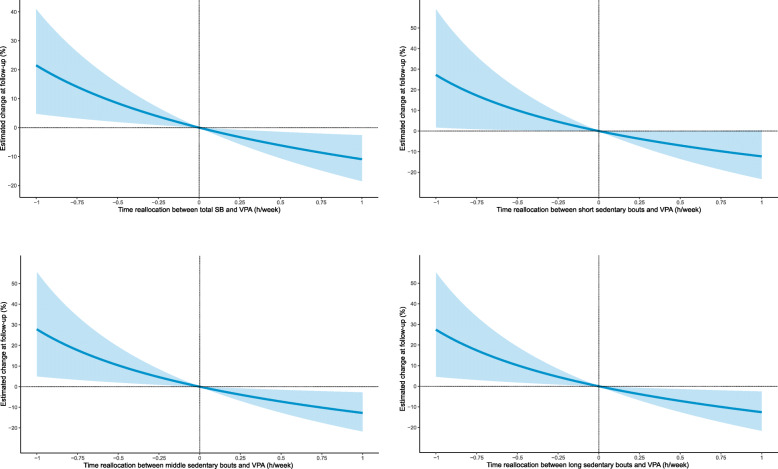
Estimated changes in visceral adipose tissue after reallocations of time from total and bouted sedentary behavior in favour of vigorous physical activity. SB – sedentary behavior, VPA – vigorous physical ativity

The prospective compositional associations between the changes in movement behaviors and adiposity indicators are displayed in Table 2. Relative to the remaining movement behaviors, the increase in time spent in middle sedentary bouts was significantly associated with higher FM% (*β*_ilr1_ = 0.27, 95% CI: 0.02 to 0.53) at follow-up. Moreover, the increase in time spent in VPA was associated with lower VAT within the 4-part (*β*_ilr1_ = − 0.30, 95% CI: − 0.52 to − 0.08) and 6-part (*β*_ilr1_ = − 0.30, 95% CI: − 0.54 to − 0.05) waking-time compositions. The estimated changes in the adiposity indicators associated with time reallocation between SB and PA of different intensities are presented in Table 3 and Fig. 1. Favorable adiposity status was associated with substituting SB with PA only for VAT. Specifically, substituting 15 min of total SB or short, middle, and long sedentary bouts with an equivalent amount of VPA was associated with a reduction in VAT of 3.3% (95% CI: 0.8 to 5.7), 3.8% (95% CI: 0.03 to 7.4), 3.9% (95% CI: 0.8 to 6.9), and 3.8 (95% CI: 0.7 to 6.8), respectively.

**Table 2:** Compositional robust regression model estimates for the adiposity markers of 5-year changes

	Fat mass (%)			Fat mass index (kg/m^2^)			Visceral adipose tissue (cm^2^)		
	*β*_ilr1_	95% CI	*p*-value	*β*_ilr1_	95% CI	*p*-value	*β*_ilr1_	95% CI	*p*-value
**Model based on the 4-part composition**									
SB (h/week)	0.06	(−0.30, 0.41)	0.757	0.03	(−0.39, 0.45)	0.888	0.48	(−0.27, 1.23)	0.209
LPA (h/week)	0.00	(−0.41, 0.41)	0.990	0.03	(−0.46, 0.52)	0.890	−0.25	(−1.09, 0.59)	0.561
MPA (h/week)	0.00	(−0.18, 0.19)	0.977	0.01	(−0.22, 0.25)	0.910	0.07	(−0.34, 0.48)	0.735
VPA (h/week)	−0.06	(−0.13, 0.02)	0.157	−0.07	(−0.17, 0.02)	0.141	**−0.30**	**(−0.52, −0.08)**	**0.010**
**Model based on the 6-part composition**									
SB short bouts (h/week)	−0.38	(−0.87, 0.12)	0.132	−0.25	(−0.82, 0.33)	0.400	0.25	(−1.01, 1.51)	0.693
SB middle bouts (h/week)	**0.27**	**(0.02, 0.53)**	**0.034**	0.24	(−0.09, 0.58)	0.152	0.22	(−0.39, 0.82)	0.482
SB long bouts (h/week)	−0.04	(−0.14, 0.07)	0.496	−0.05	(−0.17, 0.06)	0.357	0.06	(−0.12, 0.24)	0.499
LPA (h/week)	0.19	(−0.28, 0.66)	0.422	0.10	(−0.54, 0.73)	0.766	−0.32	(−1.61, 0.97)	0.620
MPA (h/week)	−0.01	(−0.18, 0.16)	0.879	0.03	(−0.20, 0.26)	0.824	0.09	(−0.35, 0.53)	0.684
VPA (h/week)	−0.04	(−0.11, 0.03)	0.278	−0.07	(−0.17, 0.03)	0.187	**−0.30**	**(−0.54, −0.05)**	**0.019**

Independent variables are expressed as the first pivot coordinate which represents the relative contribution of one behavior relative to remaining behaviors

All regression models were adjusted for age, sex, and fat mass percentage at baseline

Bold values denote significant results

*CI* confidence interval, *ilr1* isometric log-ratio (the first pivot coordinate), *LPA* light physical activity, *MPA* moderate physical activity, *SB* sedentary behavior, *VPA* vigorous physical activity

**Table 3:** Estimated changes in adiposity markers associated with isotemporal substitutions between sedentary behavior and light, moderate, and vigorous physical activity

	2 h/week from SB to LPA		1 h/week from SB to MPA		15 min/week from SB to VPA	
	Percentage change	95% CI	Percentage change	95% CI	Percentage change	95% CI
**Fat mass (%)**						
Total SB	−0.2	(−2.7, 2.3)	−0.1	(−2.3, 2.2)	−0.6	(−1.5, 0.3)
SB short bouts	3.1	(−1.5, 7.9)	1.0	(−1.2, 3.3)	−0.2	(−1.4, 1.0)
SB middle bouts	−2.8	(−6.1, 0.7)	−1.8	(−4.7, 1.1)	−0.9	(−1.8, 0.01)
SB long bouts	2.2	(−2.7, 7.2)	0.5	(−2.7, 3.7)	−0.3	(−1.3, 0.6)
**Fat mass index (kg/m**^**2**^**)**						
Total SB	0.0	(−3.0, 3.0)	0.2	(−2.6, 3.0)	−0.8	(−1.8, 0.3)
SB short bouts	1.9	(−3.6, 7.7)	1.1	(−1.9, 4.3)	−0.6	(−2.1, 0.9)
SB middle bouts	−2.7	(−7.6, 2.4)	−1.1	(−4.6, 2.5)	−1.2	(−2.5, 0.2)
SB long bouts	2.5	(−2.8, 8.0)	1.3	(−2.6, 5.4)	−0.6	(−1.9, 0.7)
**Visceral adipose tissue (cm**^**2**^**)**						
Total SB	−2.5	(−7.4, 2.7)	0.1	(−4.7, 5.1)	**−3.3**	**(−5.7, −0.8)**
SB short bouts	−2.6	(−13.6, 9.8)	0.5	(−4.0, 5.2)	**−3.8**	**(−7.4, −0.03)**
SB middle bouts	−3.7	(−12.0, 5.3)	−0.1	(−7.3, 7.7)	**−3.9**	**(−6.9, −0.8)**
SB long bouts	−3.5	(−11.6, 5.3)	0.2	(−6.5, 7.3)	**−3.8**	**(−6.8, −0.7)**

Bold values denote significant changes in adiposity status

*CI* confidence interval, *LPA* light physical activity, *MPA* moderate physical activity, *SB* sedentary behavior, *VPA* vigorous physical activity

## Discussion

Changes in adiposity and movement behaviors were revealed during the transition from childhood to adolescence. All adiposity markers, total SB, and VPA increased, while LPA and MVPA decreased. A compositional prospective association between the changes in the proportion of time spent in middle sedentary bouts and FM% in adolescence was found. Moreover, a change in VPA was associated with VAT in adolescence. Favorable changes in adiposity status were identified when the amounts of time spent in total SB and sedentary bouts of different durations were substituted with VPA.

Our findings on the age-related changes in adiposity and movement behaviors are in accordance with the available evidence [36–38]. We found that the increase in the proportion of time spent in total SB was predominantly caused by an increase in the time spent engaging in prolonged SB. Similar results were observed in 5991 participants included in a pooled analysis of longitudinal data from the International Children’s Accelerometry Database [20]. Moreover, the proportion of time spent in middle and long sedentary bouts increased at the expense of LPA and MPA in the current study. This implies that SB becomes less fragmented, as it is probably less interrupted by brief bouts of PA. This assumption might be supported by the longitudinal analysis performed by Janssen and colleagues [19], who found a decrease in the frequency of sedentary breaks and an increase in the sedentary bout duration across childhood and adolescence.

One possible explanation for the decrease in sedentary fragmentation could be a change in movement behaviors associated with the transition to secondary school [39]. As most school time corresponds to SB [40, 41] and considering the SB patterns during lessons (i.e., prolonged uninterrupted sitting), it can be assumed that the increase in prolonged SB is caused mainly by an increase in school-based SB. Another contributor to prolonged SB could be the increased time spent traveling to school in a car or bus. Adolescents are probably more likely to commute to schools located farther away from their place of residence compared with children [42]. Likewise, an age-related decrease in non-organized leisure time activities may contribute to the prolonged SB across childhood and adolescence [43]. If further longitudinal studies confirm these assumptions, interventions targeting the interruption of prolonged sitting during school and leisure time should be implemented into public health strategies.

The change in SB patterns during the transition from childhood to adolescence could have several health implications. According to this study, an increase in the proportion of middle sedentary bouts of the 10–29 min duration (relative to the remaining movement behaviors) is prospectively associated with adiposity in adolescence. Similarly to our findings, Mann and colleagues [44] showed that an increase in sedentary fragmentation expressed as an increase of one bout per sedentary hour per year was significantly associated with a decrease in BMI and FMI between 9 and 12 years of age. It seems that breaking down prolonged uninterrupted sedentary periods by sporadic PA might be an appropriate strategy for optimizing movement behaviors in the pediatric population. However, the ability to compare our findings with those previously published is limited, as, to the best our knowledge, the current study is the first to investigate prospective associations between SB patterns and adiposity using the CoDA approach. Despite this limitation, it appears that changes in SB patterns play an important role in the accumulation of adipose tissue in adolescence. Future studies are needed to identify the potential determinants of SB pattern changes, as this was beyond the scope of the present study.

We emphasize that an increase in time spent in VPA is associated with lower central adiposity in adolescence. This is in accordance with the review by Gralla and colleagues [45], who suggested that VPA is a stronger predictor for central adiposity than other intensities of PA. In light of this finding, we suggest one possible strategy to reduce adiposity is interrupting prolonged SB by bouts of VPA. This hypothesis is supported by our results from the isotemporal substitution analysis, which indicate an improved adiposity status when 15 min of total SB or all sedentary bouts are reallocated in favor of VPA. However, it is questionable whether that change could be sustainable, as it would mean more than doubling the amount of time spent in VPA in our sample. An alternative strategy may be interrupting SB by very short bouts (i.e., < 1 min) of VPA. This specific point could not be addressed in the current study, as the movement behaviors were assessed using a 60 s sampling interval. However, the findings from an experimental study conducted in children with overweight and obesity [46], for whom VPA may be difficult to achieve, support this strategy by showing an acute improvement in metabolic markers in response to interrupting SB with very short bouts of activity of lower intensity.

The current study has several strengths. First, this is the only study that considers the compositional nature of movement behavior data in analyzing the prospective associations between changes in SB patterns and adiposity. The utilization of the CoDA approach allows the control of regression models for all movement behaviors (i.e., using the set of the first pivot coordinates in the models) and the avoidance of biased estimates due to multicollinearity. Second, the device-based assessment of both adiposity markers and movement behaviors provides reliable and valid data. Finally, the SB patterns were analyzed as well, which allowed the accurate differentiation of prolonged SB from total SB.

There are also limitations within this study. Our findings should be interpreted with caution, as the study is limited to movement behaviors. Omitting time spent asleep may lead to biased estimates of regression analysis. Another source of bias relates to analyzing SB without distinguishing body postures (i.e., the differentiation of sedentary postures from standing). Thus, longitudinal CoDA-based studies based on a 24-h wear time protocol and the combination of intensity- and posture-specific assessments of movement behaviors are warranted to analyze the associations between SB patterns and adiposity across childhood and adolescence. Although the regression models were adjusted for several confounding factors, there are still potential endo- and exogenous determinants of adiposity that have not been considered. Since youth movement behaviors are characterized by their intermittent nature, the 1-min sampling interval used in this study could lead to the underestimation of high-intensity PA and may not capture all breaks in SB [47]. Another limitation is the relatively high loss of participants. However, a sensitivity analysis (Table S1) confirmed no significant differences in outcome variables between participants included and excluded from the study. Finally, our findings are not fully generalizable due to the small sample size and the higher proportion of girls. These limitations did not allow us to focus on the sex differences in associations between longitudinal changes in SB patterns and adiposity. Further research with larger samples is warranted to explore the longitudinal compositional associations separately for boys and girls.

## Conclusion

The present study revealed age-related changes in adiposity markers and movement behaviors. The increase in prolonged SB at the expense of PA implies that unhealthy time-use patterns develop during the transition from childhood to adolescence. Relative to the remaining movement behaviors, an increase in the proportion of time spent in prolonged SB over this transition period was associated with greater adiposity in adolescence, while an increase in VPA was associated with reduced central adiposity. Substituting total SB with VPA appears to be an appropriate target for intervention strategies, as high-intensity PA was associated with favorable adiposity status. These findings may help design more effective interventions to prevent an unhealthy gain in adiposity during the transition from childhood to adolescence.

## Supplementary Information

**Additional file 1: Table S1.** Characteristics of included and excluded participants.

## Data Availability

The dataset analyzed during the current study is available in the Figshare repository (10.6084/m9.figshare.14260541).

## Abbreviations

BMI: Body mass index
CI: Confidence interval
CoDA: Compositional data analysis
cpm: Counts per minute
FM%: Fat mass percentage
FMI: Fat mass index
*ilr*: Isometric log-ratio
LPA: Light physical activity
MPA: Moderate physical activity
MVPA: Moderate-to-vigorous physical activity
PA: Physical activity
SB: Sedentary behavior
VAT: Visceral adiposity tissue
VPA: Vigorous physical activity
WHO: World Health Organization
