# Supplementary material for: Changes in sedentary behavior patterns during the transition from childhood to adolescence and their association with adiposity: a prospective study based on compositional data analysis
Source: Arch Public Health. 2022 Jan 4;80:1. doi: 10.1186/s13690-021-00755-5 (PMC8725475; doi:10.1186/s13690-021-00755-5)
Supplement: Supplementary file 1 — Additional file 1: Table S1. Characteristics of included and excluded participants. [file 13690_2021_755_MOESM1_ESM.docx]

**Table S1.** Characteristics of included and excluded participants

|  |  | **Included**  **(*n* = 88)** | |  | **Excluded**  **(*n* = 223)** | |  | *p*-value^a^ |
| --- | --- | --- | --- | --- | --- | --- | --- | --- |
|  |  | Mean | SD |  | Mean | SD |  |  |
| Sex (% of girls) |  | 61.4 |  |  | 57.8 |  |  | 0.570 |
| Height (cm) |  | 137.4 | 7.3 |  | 140.6 | 7.5 |  | **<0.001** |
| Weight (kg) |  | 32.3 | 7.4 |  | 34.9 | 7.8 |  | **0.006** |
| BMI *z*-score |  | 0.18 | 1.19 |  | 0.42 | 1.14 |  | 0.115 |
| Fat mass (%) |  | 16.2 | 8.3 |  | 17.8 | 8.0 |  | 0.094 |
| Fat mass index (kg/m^2^) |  | 2.9 | 2.1 |  | 3.3 | 2.1 |  | 0.115 |
| Visceral adipose tissue (cm^2^) |  | 27.4 | 23.4 |  | 32.8 | 24.7 |  | 0.084 |
| SD – standard deviation.  ^a^ Differences between groups were tested using the one-way analysis of covariance adjusted for age and sex at baseline; the chi-squared test to compare of proportions in the sex category.  Bold values denote significant results. | | | | | | | | |
